# Arabic translation and validation of a pediatric sleep questionnaire to assess the prevalence of sleep-disordered breathing among Saudi pre-school children

**DOI:** 10.1186/s12887-022-03820-2

**Published:** 2023-01-31

**Authors:** Ahmed I Masoud, Rana H Mosli

**Affiliations:** 1 Department of Orthodontics, Faculty of Dentistry, King Abdulaziz University, PO Box 80209, Jeddah, 21589 Saudi Arabia; 2 Clinical Nutrition Department, Faculty of Applied Medical Sciences, King Abdulaziz University, PO Box 80209, Jeddah, 21589 Saudi Arabia

**Keywords:** Arabic, Children, SDB, Sleep, Translation, Questionnaire

## Abstract

**Objectives:**

(1) To translate to Arabic a validated pediatric sleep questionnaire, (2) To assess the validity and reliability of the translated questionnaire, and (3) To assess the prevalence of sleep-disordered breathing (SDB) among a group of pre-school children in Jeddah, Saudi Arabia.

**Methods:**

Using forward and back-translation, a set of 6 hierarchically arranged questions that comprise the Gozal sleep questionnaire was translated into Arabic. Validity was assessed using face validity and content validity for consistency and clarity, using both item-level and scale-level content validity indices (I-CVI, S-CVI). Consent forms were sent to 1783 mothers recruited from 8 different pre-schools in Jeddah between October 2017 and April 2018, and 209 signed and returned the consent forms. Out of this sample, 34 mothers were contacted to assess internal consistency using Cronbach's alpha, and test-retest reliability using Interclass correlation coefficient (ICC). Finally, all 209 mothers were contacted to answer the questionnaire to obtain the prevalence of SDB.

**Results:**

Using face validity and content validity, the translated questionnaire proved to be valid with perfect I-CVI and S-CVI. Internal consistency (Cronbach’s Alpha 0.64–0.89) and test-retest reliability (ICC=0.87, *p*<0.001) showed the translated questionnaire to have good to favorable reliability. Depending on the severity of SDB, the prevalence of SDB was 7.7%, 5.7%, and 3.8% for mild, moderate and severe cut-off values respectively.

**Conclusion:**

A validated pediatric sleep questionnaire to assess SDB was translated into Arabic and the translation proved to be valid and reliable. The prevalence of SDB was found to be very comparable to other areas in the world.

## Introduction

Sleep-disordered breathing (SDB), which includes snoring and sleep apnea among other sleep disorders, is an important factor for morbidity in adults and children [1–5]. In adults SDB has been linked to increased risks for hypertension, myocardial infarction, strokes, diabetes, sleepiness related accidents, and dementia [3, 6–8]. In addition to the consequences in adults, additional consequences in children include attention-deficit/hyperactivity disorder (ADHD) and other behavioral manifestations, disturbances in cognitive development, failure to thrive, and increased utilization of health care services [2, 9]. Overnight, attended, in-laboratory polysomnography (PSG) remains the gold standard for diagnosis of SDB [9, 10]. On the other hand, screening for SDB can be carried out using sleep questionnaires and thus are considerably relevant for epidemiological studies [11–14].

Pediatric sleep questionnaires are parent report tools that are concerned with the symptoms and risk factors of sleep apnea and SDB (spruyt & Gozal, 2011). Most questionnaires are too long, and a short questionnaire composed of a set of 6 hierarchically arranged questions was constructed and validated [11, 15]. Recently, Masoud et al. compared this set of 6 hierarchically arranged questions to the more commonly used Pediatric Sleep Questionnaire (PSQ) in children referred for a sleep study. They found that this set of 6 questions performed slightly better than the PSQ both in terms of correlating with the apnea-hypopnea index (AHI) and predicting sleep apnea [16].

Although SDB is widely known for affecting adults it still occurs in children. The prevalence of SDB varies in children based on the definition and ranges from 8 to 17% [5, 17–20]. More recently the prevalence of childhood obesity has gone up leading to both increased prevalence and increased awareness of pediatric sleep disorders such as obesity-related obstructive sleep apnea (OSA) [21]. In Saudi Arabia, BaHammam et al. and Wali et al. attempted to study the prevalence of SDB among adults [22, 23]. Using the Berlin questionnaire, BaHammam et al. determined that in Riyadh, Saudi Arabia, 33 to 39% were considered as high-risk patients for OSA [22, 24]. Wali et al. caried out the first population-based survey of SDB and OSA in Saudi Arabia using both a questionnaire and a PSG. Using the Wisconsin questionnaire, the authors reported the prevalence of habitual snoring in Jeddah, Saudi Arabia to be 23.5%, [23] In 2019, Baidas et al. performed the first study to determine the prevalence of SDB among children in Saudi Arabia using an Arabic version of the PSQ [25]. They found that 21% of children aged 6-12 years were at high risk of SDB in Riyadh, Saudi Arabia [25]. To our knowledge, no study has been performed to assess the prevalence of SDB among children in Jeddah, Saudi Arabia.

The objectives of the current study were: (1) To translate to Arabic a validated sleep questionnaire comprised of a set of 6 hierarchically arranged questions (6Q) proposed by Spruyt and Gozal [11]. This was accomplished out using forward-translation and back-translation methods adopted from the World Health Organization (WHO). (2) To validate the translated questionnaire by assessing face validity and content validity, and to evaluate reliability by assessing the internal consistency and test-retest reliability. (3) To use the translated questionnaire to assess the prevalence of SDB among a group of children in Jeddah, Saudi Arabia.

## Materials and methods

**Fig. 1:**
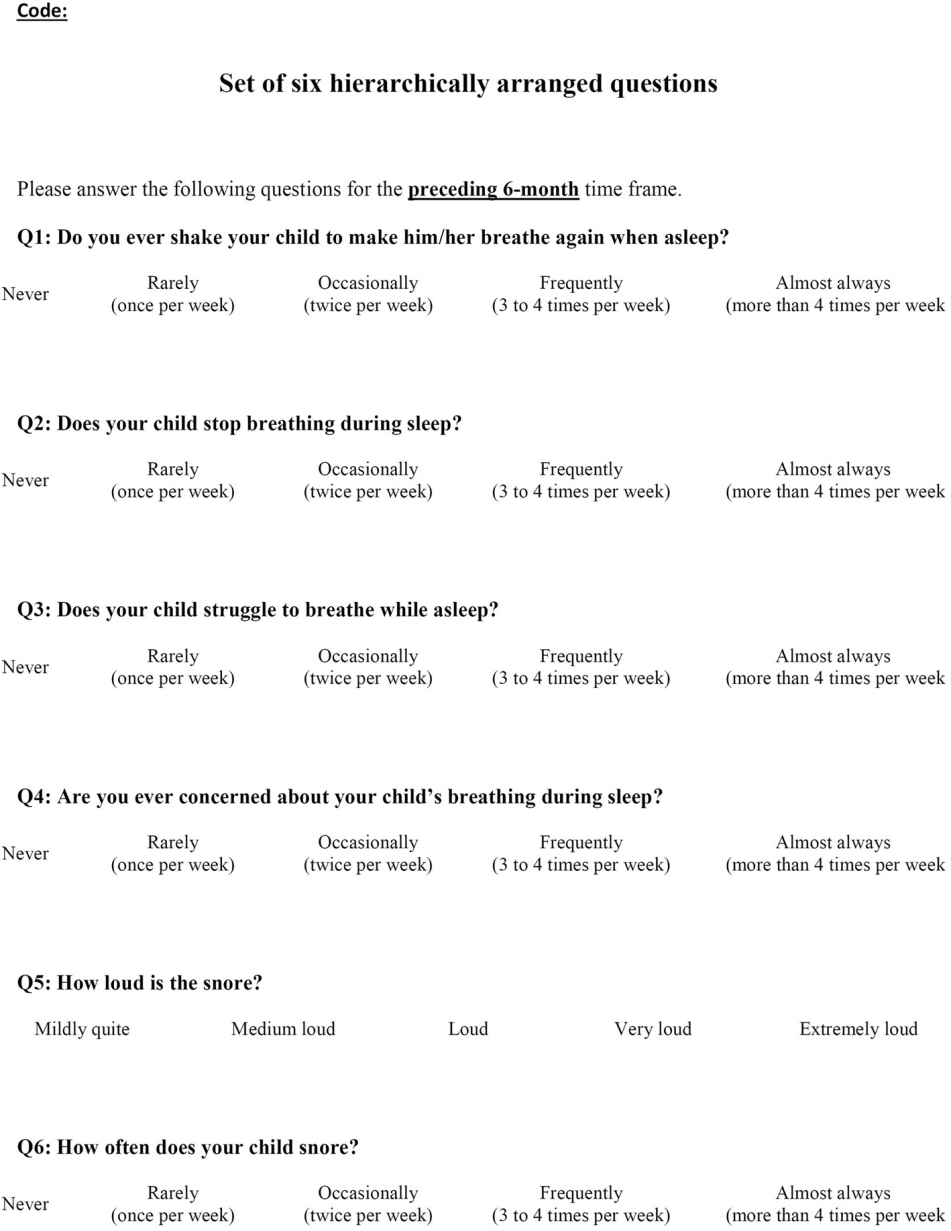
Original English version of the 6 hierarchically arranged questions that comprise the Gozal sleep questionnaire [11]

This cross-sectional study was undertaken to translate a validated set of 6 hierarchically arranged questions, that comprise the Gozal sleep questionnaire (6Q) (Fig. 1), and use it to assess the prevalence of SDB among Saudi pre-school children [11, 15]. The questions use Likert-type responses for the preceding 6 months with scores as following: “never” or “mildly quite” (0), “rarely” or “medium loud” (1), “occasionally” or “loud” (2), “frequently” or “very loud” (3), and “almost always” or “extremely loud” (4). A cumulative score is then calculated using the following formula developed by Spruyt and Gozal [11] where Q1= raw score to question1, Q2= raw score to question 2 and so on:

$$\text{A= (Q1+Q2)/2; B= (A+Q3)/2; C= (B+Q4)/2; D= (C+Q5)/2; and the final score = (D+Q6)/2}$$

Spruyt and Gozal proposed a cumulative score greater than 2.72 to be suggestive of SDB [11]. Other authors have questioned this cut-off value [15, 16]. Participants were recruited from 8 pre-schools located in different areas in Jeddah, Saudi Arabia between October 2017 and April 2018. Two pre-schools were selected randomly from each of the northern, southern, eastern, and western areas. Of the 8 pre-schools, 4 were public and 4 were private. A description of the study and consent forms were placed in the backpacks of 1783 enrolled students. Study inclusion criteria for the children were:1) Saudi or a permanent resident of Saudi Arabia, 2) age between 3 and 5 years old, 3) resides with his/her mother, 4) healthy with no serious medical problems or history of food allergies, and 5) mother is an Arabic speaker. Two hundred and nine mothers returned the signed consent forms and were contacted by research assistants over the telephone to answer the questionnaire. Written informed consent was obtained from all parents or legal guardians This study was part of a larger scale study to evaluate eating behaviors and weight status among Saudi pre-school children [26]. The protocol was approved by the research ethics committee at King Abdulaziz University (#366-16).

The study included three stages: (1) translating the English questionnaire into Arabic, (2) testing the validity and reliability of the translated Arabic questionnaire, and (3) Using the translated Arabic questionnaire to obtain the prevalence of SDB among pre-school children in Jeddah, Saudi Arabia.

### Stage 1. Translating the English questionnaire into Arabic

To translate the questionnaire, forward-translation and back-translation methods refined by the WHO were adopted [27]. To produce a final translation 6 steps were followed:

#### a. Forward-translation

**Fig. 2:**
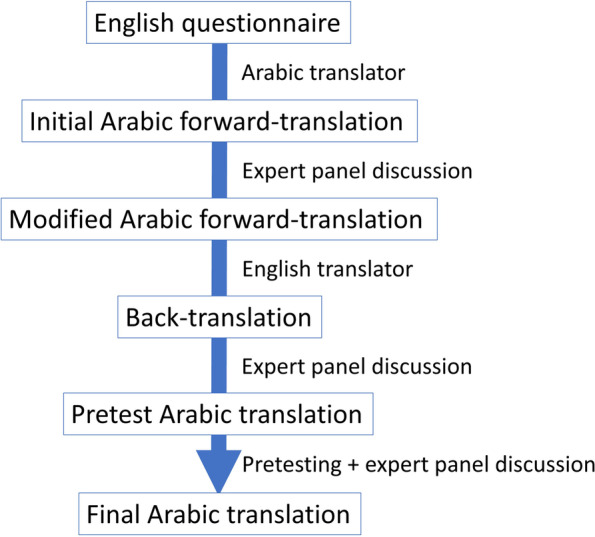
Five different translations developed during the translation process

Forward-translation was performed by the primary investigator (PI), an American trained health professional. The PI’s mother tongue is Arabic but is familiar with the terminology in the field of sleep having obtained a PhD in neuroscience and sleep from the United States. The PI emphasized conceptual rather than literal translation of words. Translations were also made to be concise avoiding long sentences with many clauses. Finally, translations were aimed to be clear and simple to consider stay-at-home mothers as the typical respondent. This resulted in an initial Arabic forward translation (Fig. 2).

#### b. Expert panel discussion of initial forward-translation

A bilingual (Arabic and English) expert panel, with Arabic as their first language, was convened by the PI to discuss the initial forward-translation. The panel included 5 American trained professionals. There were 4 health professionals with either a PhD or a Doctorate degree, and an engineer with a PhD. The PI provided the panel with reading material that familiarized them with SDB, snoring, sleep apnea, diagnosis and screening methods, and sleep questionnaires. The panel was also informed of the target audience, the way in which the questionnaire will be administered, and the conceptual framework.

The expert panel reviewed each question to identify and resolve inadequate concepts of translation, and inadequate expressions used in the translated version to ensure cultural sensitivity [28]. The panel also reviewed the questions to ensure consistency between the original and translated versions and identify discrepancies between of the questionnaires. This was carried out by questioning some expressions and words and suggesting alternatives until finally agreeing on a modified Arabic forward translation (Fig. 2).

#### c. Back-translation

Back-translation to English was performed by another health professional. The translator’s mother tongue is English but is fluent in the Arabic language having studied Arabic for 14 years in an Arabic school in Jeddah, Saudi Arabia. The translator was provided with the same reading material and information provided to the expert panel. The same concepts used by the PI for forward translation were used to create a back-translation (Fig. 2).

#### d. Expert panel discussion of back-translation

The back-translation was discussed by the same expert panel and in the same manner in which the forward-translation was discussed. The panel compared the original English version with the back-translated version to make sure the content and concept remained the same. Changes were made to the modified forward-translation and a pretest Arabic translation was composed (Fig. 2).

#### e. Pretesting and cognitive interviewing

The pretest Arabic translation was pretested on a group of 10 Saudi mothers visiting a private dental practice in Jeddah. Care was taken to include mothers of different socioeconomic levels. After the mothers filled the questionnaires, each mother was interviewed separately to discuss their responses. The mothers were asked whether they thought each question was clear, what they thought each question was asking, and to repeat each question in their own words. Additionally, the mothers were asked about certain words or phrases which the expert panel thought might be problematic, and alternative options were presented to the mothers to choose from. Finally, the mothers were asked if there were any words or phrases, other than the ones selected by the panel, that they did not understand or that could be misunderstood. A written report of all answers and problems arising during these interviews was prepared.

#### f. Expert panel discussion of pretesting results

The written report that was formulated as a result of pretesting and cognitive interviewing was presented to the same expert panel for discussion. The panel discussed all answers and issues that arose during the interviews in addition to alternative options to problematic words or phrases. Discrepancies were discussed and resolved through consensus among the expert panel to derive a final Arabic translation (Fig. 2).

### Stage 2. Testing the validity and reliability of the translated Arabic questionnaire

#### Validity

Face validity was assessed during pretesting when cognitive interviewing was performed. Face validity was determined subjectively using a dichotomous scale by asking the mothers to rate each question as “clear” or “not clear”.

Content validity was undertaken by the expert panel review which was ongoing during the translation process. Content validity was assessed at three timepoints: after forward translation, after back-translation, and after pretesting. After forward translation to ensure that adequate concepts and expression were used and consistency with the original question was maintained. After back-translation to ensure the back-translated version maintained the same concept and content as the original English version. Finally, after pretesting, each member of the panel rated each question independently in terms of “consistency with the English version” and “clarity” on a four-point scale (Table 1) [29]. The item-level content validity index (I-CVI) for each question (proportion of experts giving a question a score of either 3 or 4), and the scale-level content validity index (S-CVI) (proportion of questions on a questionnaire that achieved a score of 3 or 4 by all the experts), were calculated as will be described in the statistics section [30].

**Table 1:** Criteria for scoring questions for content validity

Score	Consistency	Clarity
1	Not consistent with English version	Not clear
2	Item needs some revision	Item needs some revision
3	Consistent but needs minor revision	Clear but needs minor revision
4	Very consistent with the English version	Very clear

#### Reliability

Reliability was conducted after content validity. The study team randomly selected 34 mothers out of the total sample of 209 to examine internal consistency and test-retest reliability. The mothers were contacted by phone to complete the questionnaire. The questionnaire had 6 questions assessing 3 constructs: apnea (questions 1 and 2), breathing difficulty (questions 3 and 4), and snoring (questions 5 and 6). To evaluate how reliably questions that were designed to measure the same construct actually did so, internal consistency was assessed. This was accomplished by determining how highly questions within the same construct were correlated and how well they predicted each other using Cronbach's alpha. In order to examine test-retest reliability, the same 34 mothers were contacted by phone approximately 2 weeks after the initial call to complete the questionnaire for a second time. The cumulative scores from the first attempt were correlated with the scores from the second attempt using Interclass correlation coefficient (ICC).

### Stage 3. Using the translated Arabic questionnaire to obtain prevalence of SDB among pre-school children in Jeddah, Saudi Arabia

As previously described, the 209 mothers who returned the signed consent forms were contacted by research assistants over the telephone to complete the questionnaire. Answers were tabulated and a cumulative score for each questionnaire was calculated.

### Statistical analysis

Statistical analysis was performed using IBM SPSS version 20. Face validity was assessed for each question separately. This was carried out by adding up the number of mothers rating each question as “clear” out of the 10 mothers the translated questionnaire was pretested on. Prior to the calculation of CVI, the consistency and clarity from the expert rating was recorded as 1 (score of 3 or 4) or 0 (score of 1 or 2) [30]. To calculate the I-CVI for each question, the number of experts giving a score of either 3 or 4 was divided by the total number of experts. To calculate the S-CVI, the total number of questions with a score of either 3 or 4 from all experts combined was divided by the total number of questions (S-CVI with universal agreement) [30].

Cronbach’s alpha was used to assess internal consistency of the raw scores within each construct and among all questions combined. Cronbach’s alpha values of at least 0.60 are considered “good”, while values of at least 0.70 are considered “favorable” [31, 32]. The cumulative scores of the sleep questionnaires were calculated using the formula described previously. Interclass correlation coefficient (ICC) of the questionnaire cumulative scores from the 34 mothers who answered the questionnaire twice was used to examine test-retest reliability. Statistical significance for all analysis was noted at α = 0.05.

To obtain the prevalence of SDB, the questionnaire cumulative scores from all 209 questionnaires were tested for normality and symmetry using Shapiro-Wilk test for normality and the average and spread were calculated accordingly. The prevalence of SDB was calculated using cumulative score cut-off values of ≥1.5, ≥2, and ≥2.5 to define different SDB severities.

## Results

Out of 1783 consent forms that were sent, 209 mothers returned the signed consent forms giving a response rate of 11.7%. After forward and back-translation, face validity was assessed during the pretesting stage. For questions 2 to 6, all mothers (10 out of 10) agreed on rating each question as “clear”. While 8 out of 10 mothers rated question 1 as “clear”, 2 out of 10 mothers thought the first question was referring to rocking the child to go back to sleep and suggestions on how to modify the question were taken from the mothers.

**Fig. 3:**
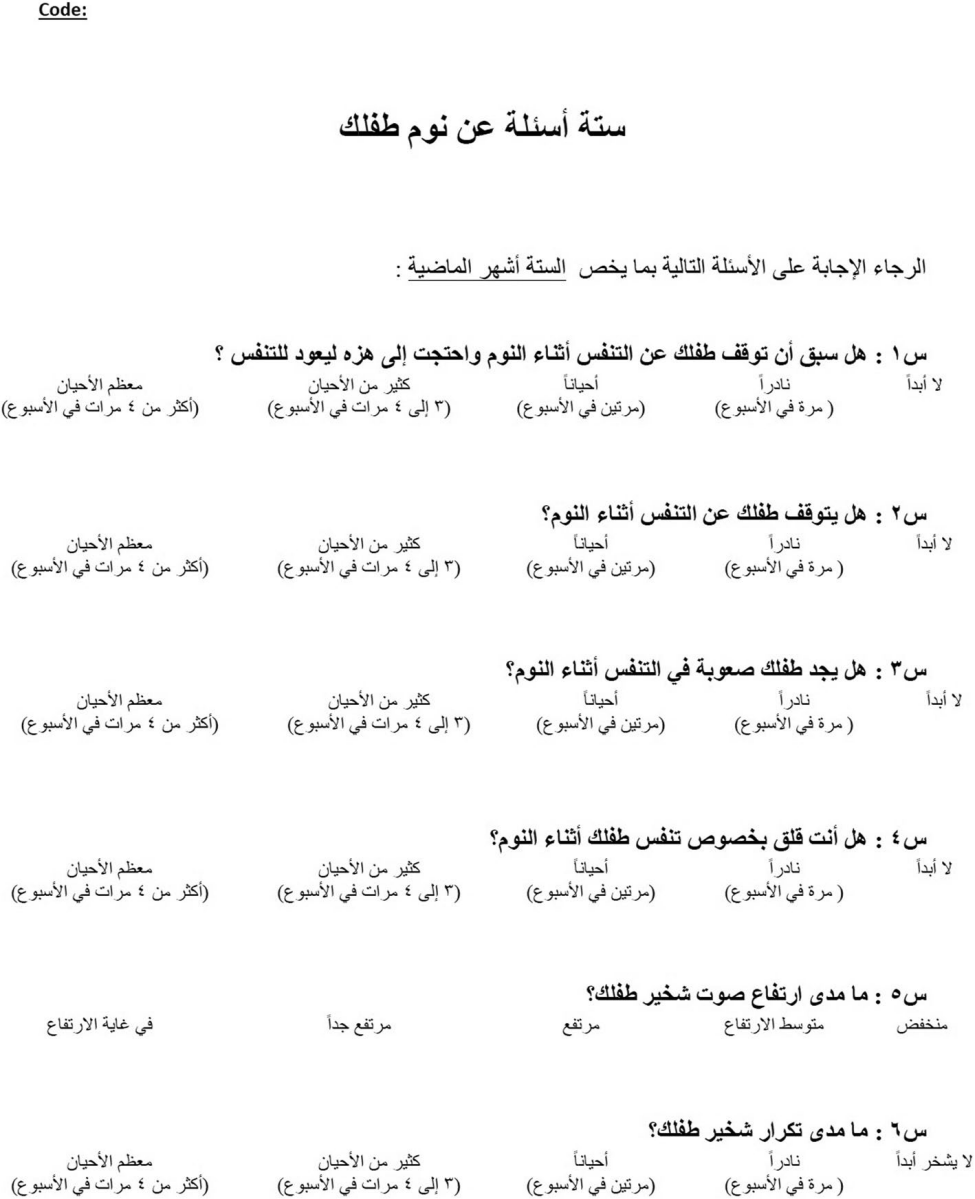
Final Arabic translation of the 6 hierarchically arranged questions that comprise the Gozal sleep questionnaire [11]

Since content validity evaluation was ongoing, modifications were made accordingly. The panel did agree with the mothers in the pretesting group that the first question could be misunderstood for rocking the child to go to sleep. After pretesting, the expert panel modified some words based on the alternative options presented to the pretest group. Additionally, based on the face validity and the ongoing content validity results, the expert panel modified the first question translation to emphasize that “shaking the child to make him/her breathe again” was after the child has stopped breathing during sleep, and modifications were done accordingly in the final Arabic translation (Fig. 3). Subsequently, content validity was assessed for the final Arabic version and Tables 2 and 3 show the content validity results, both I-CVI and S-CVI, for consistency and clarity respectively.

**Table 2:** Content validity index for consistency with the English version

Question	Expert 1	Expert 2	Expert 3	Expert 4	Expert 5	Experts in agreement	**I-CVI**
1	1	1	1	1	1	5	1.0
2	1	1	1	1	1	5	1.0
3	1	1	1	1	1	5	1.0
4	1	1	1	1	1	5	1.0
5	1	1	1	1	1	5	1.0
6	1	1	1	1	1	5	1.0
						**S-CVI**	1.0

**Table 3:** Content validity index for clarity

Question	Expert 1	Expert 2	Expert 3	Expert 4	Expert 5	Experts in agreement	**I-CVI**
1	1	1	1	1	1	5	1.0
2	1	1	1	1	1	5	1.0
3	1	1	1	1	1	5	1.0
4	1	1	1	1	1	5	1.0
5	1	1	1	1	1	5	1.0
6	1	1	1	1	1	5	1.0
						**S-CVI**	1.0

Cronbach’s alpha values for all factors ranged from 0.64 to 0.89 indicating good to favorable internal reliability. Cronbach’s alpha results within each construct and among all questions combined are shown in Table 4. The ICC for questionnaire cumulative scores obtained from the 34 mothers for test-retest reliability was 0.87 (*p* < 0.001) indicating good reliability.

**Table 4:** Internal consistency reliability for sleep questionnaire factors (*n*=34)^a^

**Factor**	**Cronbach’s Alpha**
Construct 1: apnea (questions 1 and 2)	0.89
Construct 2: breathing difficulty (questions 3 and 4)	0.79
Construct 3: snoring (questions 5 and 6)	0.64
All items	0.80

^a^Internal consistency reliability of factors was estimated by Cronbach’s Alpha. Cronbach’s alpha values of 0.70 or higher are favorable

**Fig. 4:**
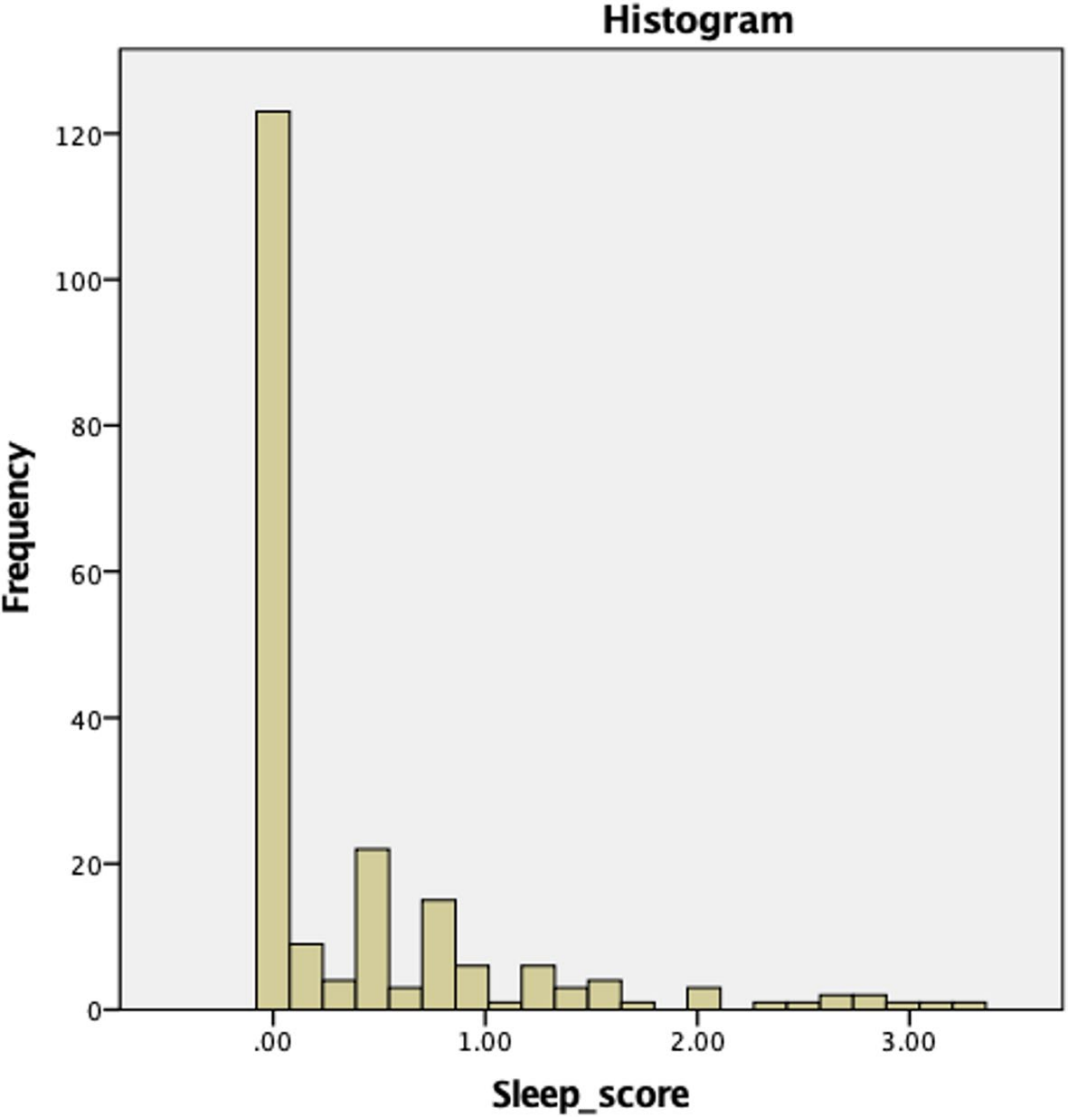
Histogram for questionnaire cumulative scores showing the data to be not normally distributed and skewed to the right

Shapiro- Wilk test for normality for all 209 questionnaire cumulative scores showed the data to be not normally distributed (*p* < 0.001). Additionally, the histogram in Fig. 4 shows the data to be skewed to the right and thus nonparametric statistics were used. The median cumulative score was 0 with an interquartile range of 0 – 0.5. The number of subjects with a cumulative score ≥1.5 was 16 (7.7%). The number of subjects with a cumulative score ≥2 was 12 (5.7%). Finally, the number of subjects with a cumulative score ≥2.5 was 8 (3.8%).

## Discussion

An essential step towards assessing the prevalence of a disorder in a population is to have a validated screening tool in the population’s language. When developing a screening tool in a different language, an important task is the actual conduct of the translation [33]. during the process of translation the emphasis should be more on thematic or conceptual translation rather than literal translation [33]. Additionally, the cultural context can be different in the translated language, therefore questionnaires should be subjected to psychometric evaluation [33]. This paper reports the process of translating and validating a pediatric sleep questionnaire comprised of a set of 6 hierarchically arranged questions (6Q) proposed by Spruyt and Gozal to the Arabic language. Afterwards, the translated questionnaire was used to assess the prevalence of SDB among a group of children in Jeddah, Saudi Arabia.

The total sample size was 209 which is smaller than previous studies [5, 17, 19, 20, 25]. The response rate at 11.7% was also relatively low compared to other studies [5, 17, 25, 34]. The current study was part of a larger scale study where mothers were asked to answer more than 60 questions [26]. The large number of overall questions might have refrained mothers from answering the questionnaires. In the study by Baidas et al., 1600 questionnaires were distributed and 1350 completed and returned the questionnaires giving a much larger total sample with a greater response rate [25]. However, this is the first study of its kind in Jeddah, Saudi Arabia and can be considered as a pilot study for future studies.

Initial face validity was perfect for all questions except for question 1 which was then modified by the expert panel. There was consensus among the expert panel that all 6 questions were “clear” and “consistent”. Polit and Beck recommend an I-CVI of 1.0 for an expert panel of 5 or fewer judges [30]. In the current study there were 5 experts and the I-CVI was 1.0 for all questions in terms of consistency and clarity. Tables 2 and 3 show that I-CVI and S-CVI met satisfactory levels, and thus the questionnaire achieved a satisfactory level of content validity. The results for internal consistency and test-retest reliability showed good reliability and thus also achieving a satisfactory level of reliability.

When the 6Q questionnaire was developed, a cumulative score of >2.72 was suggested to identify SDB (AHI >3) [11]. However, other authors suggested a cumulative score lower than 2.72 to be more sensitive in identifying SDB [15, 16]. Kadmon et al. suggested a cumulative score of ≥1 to identify an AHI ≥5 [15]. More recently, Masoud et al. recommended cumulative scores of ≥1.5, ≥2, and ≥2.5 to identify mild OSA (AHI≥1.5), moderate OSA (AHI≥5), and severe OSA (AHI≥10) respectively [16]. In the current study, the cumulative score cut-off values recommended by Masoud et al. were used and the prevalence of SDB was 3.8%, 5.7%, and 7.7% for mild, moderate, and severe cut-off values respectively.

As previously mentioned, the prevalence of SDB among children In Jeddah, Saudi Arabia has not been assessed. In Riyadh Saudi Arabia, Baidas et al. used the PSQ and found that 21% of children aged 6-12 years were at high risk of SDB [25]. In other parts of the world. a large population-based study in England was conducted where pre-school children were followed for 6 years. The authors reported that for different ages the percentage of children with habitual snoring ranged from 9.6% to 21.2% and children who always snored ranged from 3.6% to 7.7% [13]. While in Germany, a study on primary school children with a mean age of 9.6 years showed that children who snored frequently or always accounted for 10% of the study sample. The researchers used an extended version of the Gozal sleep questionnaire that was used in the current study but no cumulative scores were calculated [19]. In comparison, the number of children who snored “frequently” or “always” in the current study was 13 (6.2%). In the United States, Rosen et al. assessed the prevalence of SDB in 8- to 11-year-old children using both a questionnaire and a home limited channel sleep study. They found that 17% of children snored loudly once or twice per week which is a much lenient definition compared to other studies. However, the results of their home sleep studies showed that a maximum of only 4.7% had SDB [20]. Another study in the United States found that 10.5% of children 4 to 11 years of age snored loudly frequently or almost always, and 3.8% had apneas witnessed by their parents [5]. In the present study, using different cut-off values to include different SDB severities, the prevalence of SDB ranged from 3.8% - 7.7% which is quite comparable to studies in different countries. Additionally, the Gozal sleep questionnaire was used in the current study instead of the PSQ since it has shown stronger AHI correlation and predictive value compared to the PSQ [16].

Our study was the first to translate and validate the Gozal sleep questionnaire and the first to assess SDB among children in Jeddah, Saudi Arabia. Having a valid and reliable Arabic assessment tool for SDB is needed in order to design and implement comprehensive prevention and intervention programs in Saudi Arabia and other Arabic speaking countries. Children with SDB are at an increased risk for numerous health issues and poorer quality of life especially since the rates of being overweight or obese among children in Saudi Arabia, and the Middle East in general, continue to be problematic [35, 36]. Accurate assessment and effective treatment of SDB among children can help improve short- and long-term health outcomes and the quality of life among youths.

The findings of the current study must be seen in light of two notable limitations. The first being the sample size used to assess the prevalence of SDB was relatively small. However, as mentioned above , this is the first study to assess the prevalence of SDB among children in Jeddah, Saudi Arabia and can be regarded as a pilot study for future studies. Nevertheless, the sample size used to assess the validity and reliability of the questionnaire was considered adequate. The second limitation was that validity was assessed using face validity and content validity but not construct validity. Ideally, the translated questionnaire should have been validated using sleep studies which are considered the gold standard for sleep disorder diagnosis. Having said that, Spruyt and Gozal dedicated a study to compare the results of the English version of the questionnaire to the results of overnight sleep studies and showed it to be a good screening tool for children at high risk for SDB [11]. The current study accepted the English version of the questionnaire as a validated tool and used it to assess content validity of the newly translated questionnaire.

## Conclusion

In summary, a validated pediatric sleep questionnaire to assess SDB was translated into the Arabic language using forward-translation and back-translation. The translated questionnaire proved to be valid with good to favorable reliability. The translated questionnaire was used to assess the prevalence of SDB among a group of pre-school children in Jeddah, Saudi Arabia and found it to be 7.7%, 5.7%, and 3.8% for mild, moderate, and severe cut-off values respectively, very comparable to other areas in the world. The is the first study of its kind in Jeddah, Saudi Arabia and a larger-scale future study is needed to include a larger sample from the different regions of the country, and to include children of different age groups.

## Data Availability

The datasets used and/or analysed during the current study are available from the corresponding author on reasonable request.
